# Differential nucleobase protection against 5-fluorouracil toxicity for squamous and columnar cells: implication for tissue function and oncogenesis

**DOI:** 10.1007/s10637-015-0259-x

**Published:** 2015-07-01

**Authors:** John P. Vanden Heuvel, Jerry T. Thompson, Prajakta Albrecht, Donald Mandetta, Harry Kamerow, John P. Ford

**Affiliations:** Department of Veterinary and Biomedical Sciences and Center for Molecular Toxicology and Carcinogenesis, Penn State University, 325 Life Sciences Building, University Park, PA 16802 USA; INDIGO Biosciences, Inc., 1981 Pine Hall Road, State College, PA 16801 USA; Mount Nittany Medical Center, 1850 East Park Avenue, State College, PA 16803 USA; Asymmetric Therapeutics LLC, 141 Main St., PO Box J, Unadilla, NY 13849 USA

**Keywords:** 5-fluorouracil, Cancer chemotherapy, Pyrimidine salvage, Toxicity prevention, Preclinical studies, Oncogenesis

## Abstract

*Purpose* The goal of these studies was to test if local excess of a normal nucleobase substrate prevents the toxicity of protracted 5FU exposure used in human cancer treatment. *Methods* Messenger RNA expression studies were performed of 5FU activating enzymes in human colon cancer cells lines (CaCo-2, HT-29), primary human gingival cells (HEGP), and normal esophageal and gastric clinical tissue samples. Excess nucleobase was then used in vitro to protect cells from 5FU toxicity. *Results* Pyrimidine salvage pathways predominate in squamous cells of the gingiva (HEGP) and esophageal tissue. Excess salvage nucleobase uracil but not adenine prevented 5FU toxicity in HEGP cells. Pyrimidine *de novo* synthesis predominates in columnar Caco-2, HT-29 and gastric tissue. Excess nucleobase adenine but not uracil prevented 5FU toxicity to Caco-2 and HT-29 cells. *Conclusion* The directed application of the normal nucleobase uracil to the squamous cells of the oral mucosa and palms and soles together with the delivery of the normal nucleobase adenine to the columnar cells of the GI tract may enable the safe delivery of higher 5FU dose intensity. These results also suggest a feature of tissue function where squamous cells grow largely by recycling overlying tissue cell components. Columnar cells use absorbed surface nutrients for *de novo* growth. A disruption of this tissue function can result in growth derived from an underlying nutrient source. That change would also cause the loss of the region of cell turnover at the tissue surface. Subsequent cell proliferation with limiting nutrient availability could promote oncogenesis in such initiated tissue.

## Introduction

5-Fluorouracil (5-FU) and its pro-drugs are a component of the standard therapy for a variety of malignancies. However, the toxicity, in particular to the gastrointestinal tract and skin of hands and feet, limits the overall utility of these drugs [1]. 5-FU rapidly enters the cell using the same facilitated transport mechanisms as uracil, including the nucleobase transporters SLC29A2 [2] and SLC22A7 [3]. 5-FU is converted intracellularly to several active metabolites by three routes [4]: the 5-phosphoribosyl 1-pyrophosphate (PRPP)-mediated conversion of 5-FU to 5-fluoro-uridine-monophosphate (FUMP) by orotate phosphoribosyl transferase (OPRT)/Uridine monophosphate synthetase (UMPS); sequential conversion of 5-FU to FUMP by uridine phosphorylase (UPP1) and uridine kinase (UCK2), and; the sequential conversion of 5-FU to 5-fluoro-deoxy-uridine-monophosphate (FdUMP) by thymidine phosphorylase (TYMP) and thymidine kinase (TK1). The antitumor activity results from inhibition of thymidylate synthase (TS) by FdUMP, as well as from incorporation of 5-FU metabolites into RNA and DNA. Only a small part of the 5-FU dose is activated via these routes, as 80–90 % of the administered dose in humans is degraded to 5, 6 dihydrofluorouracil (DHFU) by dihydropyrimidine dehydrogenase (DPYD).

The toxicity and efficacy of 5-FU is highly variable and is dependent on the expression and polymorphisms of the detoxification and activation enzymes. For example, the Gly213Ala polymorphism in UMPS/OPRT is predictive of GI toxicity following 5-FU chemotherapy [5], a finding that may be relevant for the present study. DPYD mutations are associated with altered 5-FU toxicity (6). The inter-individual differences in expression of 5-FU metabolism enzymes in addition to functional protein differences further complicates the prediction of the likelihood of toxicity to 5-FU [6]. In vitro, the expression of enzymes such as DPYD, TYMS, UCK2, and UMPS influence sensitivity to 5-FU [7]. The use of oral 5-FU results in irregular absorption with marked intra- and inter-individual differences due to the variable activity of DPYD, especially in the GI tract.

As a result of these concerns, oral 5-FU pro-drugs have been developed [1, 8]. Three commonly utilized 5-FU prodrugs are capecitabine (CAP), UFT (ftorafur (FTO) plus uracil), and S-1 (FTO plus 5-chloro- 2,4-dihydroxypyridine and potassium oxonate) [1]. CAP is converted into 5-FU in a three-stage process. In an alternate effort to improve the therapeutic index of the 5-FU, UFT, a mixture of the FTO and uracil (U) in molar proportions of 1:4, has been developed. Uracil, a natural substrate for DPYD, competes with 5-FU for degradation [9]. Finally, a third alternative to improve the therapeutic index of 5-FU is S-1, a combination of a FTO and 5-chloro-2,4-dihydroxypyridine (CDHP) and potassium oxonate (OXO). CDHP is a potent and reversible inhibitor of DPYD and OXO is a competitive inhibitor of UMPS. UFT and S-1 expose the patient systemically to drug components, which may alter the therapeutic index of 5FU. Systemic uracil in UFT may compete for the activation of 5-FU in the tumor by UPP-1 and decrease anti-tumor activity. OXO in S-1 may also inhibit UMPS in the normal GI tract and decrease the synthesis of UMP and thus increase the toxicity of 5-FU.

We attempted to prevent the toxicity of 5-FU by a strategy of applying a non-competitive inhibitor, a normal substrate of the target enzyme of 5-FU, locally and in excess to the tissue cell-type subject to toxicity. Using cell culture model systems, we demonstrate that adenine, but not uracil, is effective and non-toxic in preventing 5-FU toxicity in the colon cancer cell lines (Caco-2 and HT-29). Uracil, but not adenine, is effective at preventing 5-FU toxicity and is itself non-toxic to primary gingival cells (HGEP). Differences in gene expression of pyrimidine metabolic enzymes between the tissue types may explain the differential sensitivity to nucleobase protection of 5-FU toxicity.

To sustain growth squamous cells of the oropharynyx rely predominantly on salvage of cell constituents from the overlying cells closer to the tissue surface. GI glandular cells rely predominantly on *de novo* pyrimidine synthesis from nutrients in the GI contents. This gene expression pattern difference paradoxically suggests a fundamental common feature of oncogenesis for both squamous and columnar cells of the GI tract. For normal cells of both tissues there may be a normal nutrient-driven growth away from the zone of replication at the epithelial/mesenchymal interface and towards the surface. When the normal growth directed away from the zone of replication is inadequate to meet the nutrient requirement of the tissue, both squamous and glandular cells evoke a nutritional response through neovascularization of the underlying mesenchymal layer. The consequence for both squamous and columnar cells is postulated to be growth towards, rather than away from, the zone of replication. A competition for nutrients and survival could develop at the epithelial/mesenchymal junction and leads to dysplasia and if sustained oncogenesis. Taken together, these studies show differential protection of 5-FU toxicity by nucleobases and also suggest a fundamental common characteristic of GI epithelial tissue function.

## Material and methods

### Cell culture

Caco-2, obtained from American Type Culture Collection (ATCC, Manassas, VA), were grown in DMEM supplemented with 5 ml penicillin (100 UI/ml), streptomycin (100 μg/ml), 5 ml amphotericin B (250 μg/ml) and 5 % FBS. Normal human gingival progenitor cells, cryopreserved at P2 (HGEP), were cultured as instructed by the supplier, (Zen-Bio, Research Triangle Park, NC) using the supplied media and antibiotics. Cells were seeded into 96-well tissue culture plates and treated as outlined in the figure legends. Cell viability was assessed using CellTiter-Glo® Luminescent Cell Viability Assay following the supplied protocol (Promega Corp., Madison WI). For experiments where delivery of nucleosides was by liposomes, Trans-IT TKO (Mirus, Madison WI) was used following the protocol provided for delivery of siRNA.

### Tissue samples

After obtaining informed consent, 5 paired biopsy specimens were obtained during routine upper endoscopy at the Mount Nittany Medical Center from the squamous cells lining the esophagus, above the gastroesophageal junction, as well as from the columnar cells lining the gastric mucosa, below the gastroesophageal junction. The project was presented to and approved by the Institutional Review Board at Mount Nittany Medical Center. One portion of the biopsy specimens was analyzed in part by microscopy to confirm the predicted histology. No sample revealed significant pathology. The remaining tissue was snap-frozen on dry ice and subsequently stored at −80 °C until analysis.

### Gene expression analysis

Total RNA was isolated from the tissues using TriReagent (Sigma, St. Louis, MO) according to the manufacturer’s instructions; real time quantitative PCR was performed as previously described [10–12]. The total RNA was reverse transcribed using the ABI High Capacity cDNA archive kit (Applied Biosystems, Foster City, CA). Standard curves were made using serial dilutions from pooled cDNA samples. Real-time polymerase chain reaction (PCR) was performed in the presence of SYBR green and amplified on the ABI Prism 7000 Sequence Detection System. The genes examined and primer sequences are shown in Table 1.

**Table 1:** Primer sequences

Gene name	Forward primer	Reverse primer
APRT	AGCCCAGTCCAAGCTCCT	ACACCTGAAGGCGACCC
DPYD	GATGCCCCGTGTCAGAAGAG	GGTCCCTCTTCAGTGGCATA
PRPS	CCGTGATCGCTTAGTGGAGT	GATTTCGCCACAACCACTCTG
SLC22A7	CACACTCCATCCAGCAAGG	TTGTACCCTACGGTGCTCAG
SLC29A2	CTGTGTGGGCATCCTCATGT	GATTCCGGCTCCTTCTCCAG
TK1	TCCAGTGCAGCCACAATTAC	CTGTCATAGGCATCGACGAG
TYMP	GGATTCAATGTCATCCAGAGCCCA	TGAGAATGGAGGCTGTGATGAGTG
UCK2	ACCCGGATGCCTTTGACAAT	AATACTCTGCGTGAGAGCCG
UMPS	GTTGTCAAAACTGATGCCTG	CCCCATCTACATCGATCTGC
UPP1	ACAATGATTGCCCCGTCAGA	ATGGCATAGCGGTCAGTTCC

### Statistical analysis

Differences between treatments were determined using ANOVA followed by Dunnett’s post-hoc test (JMP 7, SAS Institute, Cary NC). Significant differences were determined when *p* < 0.05. Non-linear regression and EC_50_ calculations were performed with Prism 5.0 (GraphPad Software, Inc., San Diego, CA).

## Results

**Fig. 1:**
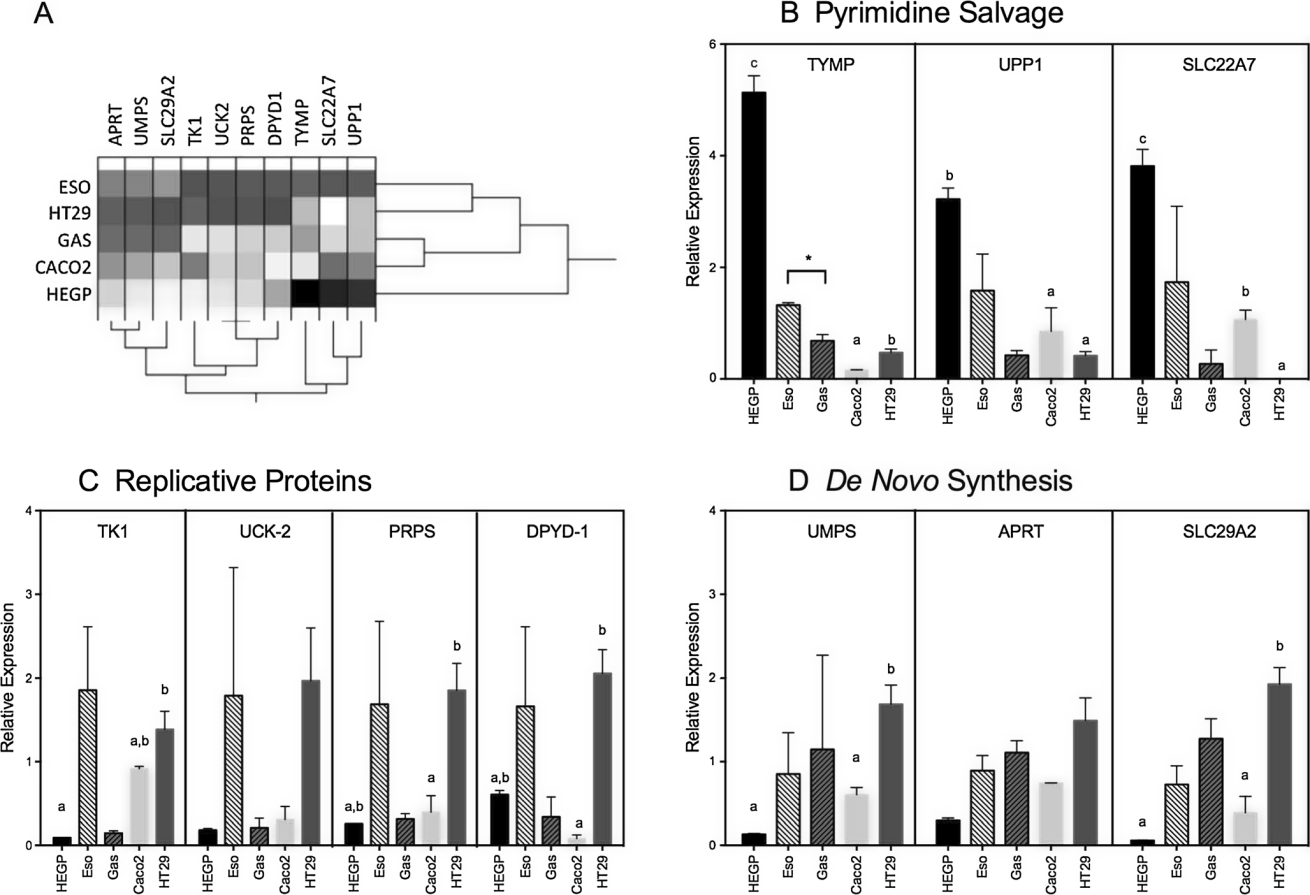
Expression of mRNA for 5-FU metabolism enzymes. The basal level of expression of messenger RNA for 5-FU metabolic enzymes in Caco-2, HT29 and HEGP cells (*n* = 3) as well as esophagus (Eso) and gastric (Gas) biopsies (5 individuals) was assessed by quantitative RT-PCR. Data is expressed relative to β-actin. The Esophogus and Gastric tissues were compared using paired *t*-test and asterisk denotes a significant difference between the samples (*p* < 0.05). For the cell lines, bars with different letters are significantly different from each other (ANOVA, followed by Tukey’s multicomparison test, *p* < 0.05)

The levels of various transcripts encoding key enzymes of 5-FU metabolism were examined in Caco-2, HEGP, HT-29 cells as well as human biopsy samples taken from squamous (esophagus, Eso) and epithelial (gastric tissue, Gas) tissues. Specifically, those enzymes as part of pyrimidine salvage (TYMP, UPP1, SLC22A7), DNA replication (TK1, UCK-2, PRPS, DPYD-1) and *de novo* nucleotide synthesis (UMPS, APRT, SLC29A2) pathways (Fig. 1) were examined. Interestingly, when the expression of each gene was examined across all the samples using hierarchical clustering, the genes organized into the three ontological pathways (Fig. 1a). The pyrimidine salvage pathways predominated in the oral and esophageal samples whereas the replicative and *de novo* synthesis pathways were expressed at a higher level in the colon cancer-derived lines. TYMP mRNA was higher in Eso versus Gas biopsies and was different in each of the three cell lines with HEGP showing the highest expression (Fig. 1b). UPP1 expression was similar between Eso and Gas samples, but was significantly higher in HEGP than in Caco-2 or HT29. A similar pattern of gene expression was seen for SLC22A7, although in this instance Caco-2 exhibited higher expression of this mRNA than did HT-29. TK1 expression was slightly different between Eso and Gas (*p* < 0.08 with one outlier) samples and was lower in HEGP relative to Caco-2 and HT-29 (Fig. 1c). Due to high variability, there were no significant differences in UCK-2 expression, although PRPS and DPYD-1 mRNA accumulated in HT-29 relative to Caco-2. For the *de novo* synthesis transcripts (Fig. 1d), the absolute mRNA levels were higher for UMPS, APRT and SLC29A2 in Gas compared to Eso biopsies, but none was significantly different. UMPS and APRT mRNA levels were lowest in HEGP progenitor cells.

**Fig. 2:**
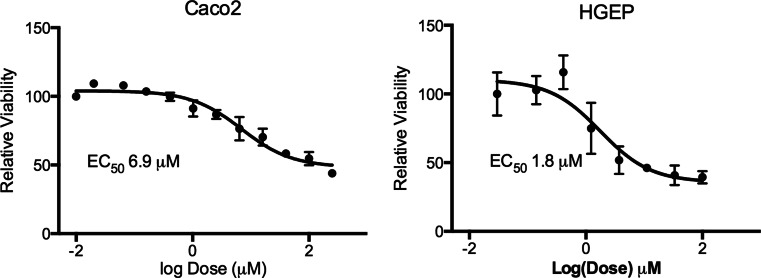
Sensitivity of Caco-2 and HGEP cells to 5-FU toxicity. Cells were plated in 96-well plates and allowed to adhere overnight. Subsequently, 5-FU was dissolved in water and added to media at various concentrations. Media was changed every day with reapplication of 5-FU. After 72 h, the cell viability was assessed and is expressed relative to the vehicle control. (Caco-2, *n* = 3; HGEP, *n* = 4)

In view of the fact that gene expression was different between pyrimidine salvage enzyme transcripts, the sensitivity to 5-FU and nucleobase competition was examined using Caco-2 cells to typify the GI tract, comprised predominantly of epithelial cells, and HGEP cells to represent oral mucosa squamous epithelial cells. Both Caco-2 and HGEP cells were treated with various concentrations of 5-FU for 72 h, with fresh media and 5-FU delivered daily (Fig. 2). The Caco-2 cell line was slightly less sensitive to 5-FU than was HGEP (EC_50_ 7 versus 2 μM, respectively). Neither cell line exhibited 100 % cell death, even after treatment for 1 week. In fact, in Caco-2 cultures a population of cells that were resistant to 5-FU emerged and cell viability increased slightly following longer-term treatment (data not shown).

**Fig. 3:**
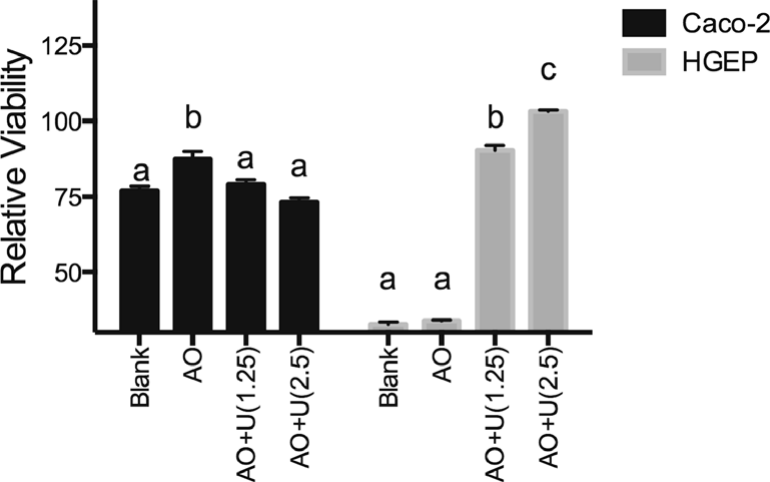
Protection of 5-FU toxicity by aqueous delivery of adenine plus orotate. Cells were plated in 96-well plates and allowed to adhere overnight. Subsequently, cells were treated with adenine plus orotate (2.5 mM each, A/O) or A/O plus uracil (A/O/U) at 1.25 and 2.5 mM in water. 5-FU was dissolved in water and added to media at 10 μM. Media was changed every day with reapplication of 5-FU and nucleosides. After 72 h, the cell viability was assessed and is expressed relative to the vehicle control. (Caco-2, *n* = 3; HGEP, *n* = 4). Within each cell line, bars with different letters are significantly different from each other (ANOVA, followed by Tukey’smulticomparison test, *p* < 0.05)

The nucleobases adenine (A), orotate (O) and uracil (U) were examined as inhibitors of the toxic effects of 5-FU. Delivery of adenine and orotate to Caco-2 cells in aqueous solution lead to a protection against 5-FU toxicity (Fig. 3). Addition of uracil to the AO solution negated the cytoprotective effects seen in Caco-2. In contrast, aqueous delivery of AO to HGEP cells had no effect in the absence of uracil, but uracil showed a dose-dependent increase in protection against 5-FU. Most mammalian cells exhibit low-affinity, equilibrative nucleoside and nucleobase transporters (ENTs, SLC29 family), while some tissues, typified by liver, small intestine and kidney, also exhibit concentrative, sodium-dependent nucleoside transport (SLC28 family) [2]. To assess the role of these transporters on uptake of the nucleobases adenine, orotate and uracil, liposomes were prepared for delivery into Caco-2 and HGEP cells. The application of the empty liposomes themselves reduced 5FU toxicity to the cells but the results with the application of uracil, adenine and orotate as 5FU protective agents were similar to those obtained by aqueous incubation with nucleobases (data not presented). Thus, delivery of the nucleobases via SLC transporters is unlikely to be responsible for the differences observed in cytoprotection of adenine, orotate and uracil from 5FU toxicity. Because of the relative simplicity of aqueous incubation, the subsequent experiments utilized this method of treatment.

**Fig. 4:**
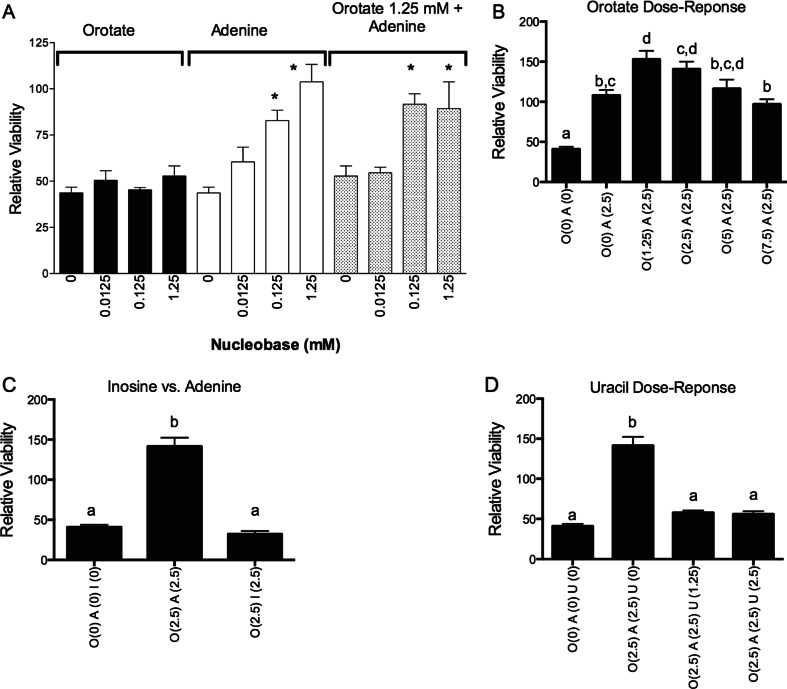
Dose–response relationships for adenine (*A*), inosine (*I*), uracil (*U*) and orotate (*O*) for protection of Caco-2 cells from 5-FU toxicity. Cells were plated in 96-well plates and allowed to adhere overnight. Subsequently, cells were treated with adenine (*A*), inosine (*I*), uracil (*U*) and orotate (*O*), in concentration as shown in parenthesis beneath each bar (in mM). 5-FU was dissolved in water and added to media at 10 μM. Media was changed every day with reapplication of 5-FU. After 72 h, the cell viability was assessed and is expressed relative to the vehicle control. (Caco-2, *n* = 3). Panel **a**, *asterisks* denote significance from respective control. Panels **b**-**d**, bars with different letters are significantly different from each other (ANOVA, followed by Tukey’smulticomparison test, *p* < 0.05)

The cytoprotective effect of aqueous delivery of adenine, orotate and uracil in Caco-2 cells was examined in more detail (Fig. 4). Adenine at 0.0125 mM through 1.25 mM resulted in progressively greater cytoprotection against 10 μM 5-FU toxicity (Fig. 4a). Addition of orotate (0–1.25 mM) did not attenuate 5-FU toxicity produced by adenine. At higher concentrations of adenine (2.5 mM), the addition of orotate at 1.25 mM may have provided enhanced protection against 5-FU toxicity in Caco-2 cells. Still higher concentrations of orotate caused progressively greater abrogation of salvage from 5-FU toxicity (Fig. 4b). As shown in Fig. 4c, the purine nucleoside inosine does not prevent 5-FU toxicity in contrast to the purine nucleobase adenine. A reversal of AO cytoprotection by uracil was seen in cells exposed to 10 μM 5-FU (Fig. 4d).

## Discussion

Differential expression of the enzymes of *de novo* synthesis versus salvage of pyrimidine nucleosides between anatomic regions may explain the differences in nucleobase specificity in preventing 5-FU toxicity. The expression of pyrimidine salvage enzymes, including TYMP, UPP1 and SLC22A7, is highest in HEGP of oral cavity-derived keratinized squamous cell tissue. HEGP cells were slightly more sensitive to 5-FU toxicity and were protected from 5-FU toxicity by uracil that can be salvaged by TYMP1 to uridine but not by adenine and orotate, nucleobases involved in *de novo* pyrimidine synthesis. The esophagus has a simple stratified squamous epithelial surface without keratin. The esophagus utilizes both salvage and *de novo* synthesis, as evidenced by the slightly higher expression of UMPS, APRT and SLC29A2 relative to the gingival cell line that has a keratin layer. The increased proliferative enzymes in the distal esophagus are consistent with tissue stress.

The comprehensive analysis of gene expression in human GI tract and Caco-2 [13] was reanalyzed to examine 5-FU metabolic enzymes (data not shown) and illustrated that Caco-2 cells most closely resemble cells of the colonic epithelium. For the Caco-2 cells of columnar origin, exposure to adenine showed a significant and dose-dependent protective effect from 5-FU toxicity. The sensitivity of Caco-2 cells to adenine cytoprotection may be dependent on the dominant role of *de novo* synthesis, in particular UMPS. The mechanism of protection by adenine may be the result of relative depletion of PRPP by APRT. The K_m_ for PRPP are similar for APRT (33 μM, [14]) and UMPS (25 μM, [15]), which indicates that in the presence of vast excess adenine relative to 5-FU, the utilization of PRPP by APRT will be favored. In addition because of pK difference, the K_m_ of UMPS for orotate (5.5 μM) is 30-fold lower than that of 5-FU (190 μM [15]). Thus, with limited PRPP availability endogenously produced orotate will be utilized by UMPS over 5-FU.

The failure of orotate as a monotherapy to prevent 5-FU toxicity probably relates to two facts: orotate with a negative charge at neutral pH is poorly taken-up by the cells. Because of its low K_m_ for UMPS, increased intracellular orotate competes successfully for PRPP and can deplete PRPP and can cause “purineless” cell death.

**Fig. 5:**
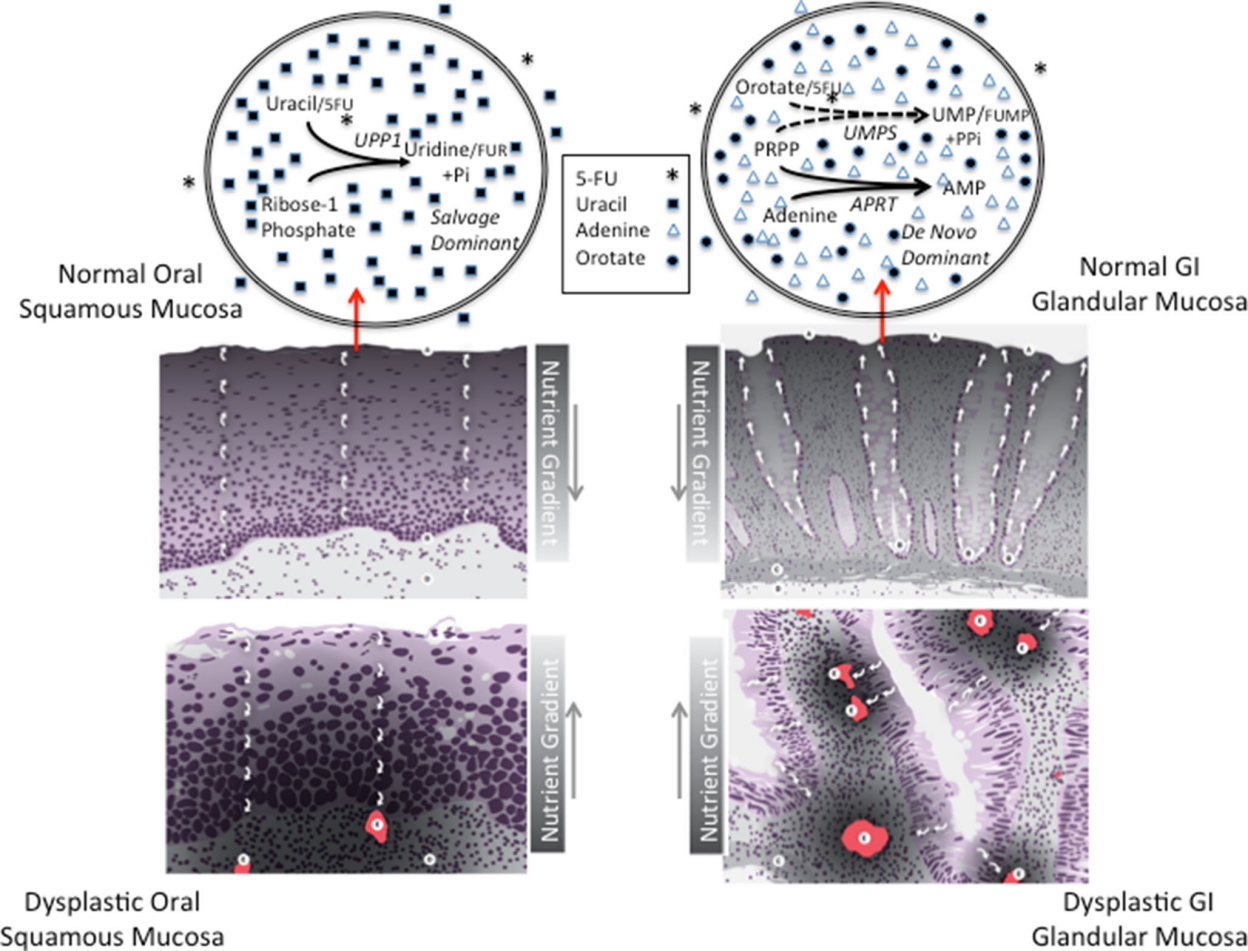
NDGP in normal and dysplastic GI squamous and glandular cells and 5-FU metabolism in normal cells. Shown in the *top panels* are the predominant pathways of 5-FU metabolism. In normal oral squamous cells salvage pathways dominate and uracil competes with 5-FU for metabolism by UPP1/TYMP. In normal GI glandular mucosa, *de novo* synthesis pathways dominate and excess exogenous adenine relatively depletes PRPP though metabolism by APRT and makes it rate limiting for UMPS and favors the lower Km orotate substrate over 5FU. The *lower panels* represent the source of nutrients and areas of cellular replication in both normal and dysplastic tissues. Labels: **A**, Zone of cell turnover; **B**, Zone of replication; **C**, muscularis mucosa layer; **D**, Mesenchymal layer; **E**, Blood vessel. *White arrows* depict route of cellular migration. The nutrient gradient is depicted with shading within the image, with darker shading representing higher levels of nutrients. The drawings are based on photographs of pathologic slides. The panels depicting dysplastic tissues ignore nutrient gradients that would originate from blood vessels in the tissue either above or below the plane of the photographed, thin pathologic specimen

Uracil enhanced 5-FU toxicity in Caco-2 cells. This result may reflect the depletion of ribose-1-phosphate by uracil, thereby increasing 5-FU concentration available for activation by UMPS in a cell type significantly dependent on *de novo* synthesis of pyrimidine nucleosides. Because of its much higher K_m_, uracil competes ineffectively with 5-FU for activation by UMPS. Taken together, these studies have shown that differences in gene expression of pyrimidine metabolic enzymes may explain the differential sensitivity to nucleobase protection of 5-FU toxicity (see Fig. 5, top panels).

Evidence for a clinical role for uracil protection from 5-FU toxicity already exists. Systemic uracil is a component of UFT that is in clinical use and UFT is well-tolerated and, in contrast to the administration of 5-FU alone, has a very low incidence of both 5-FU cutaneous toxicity (“hand-foot” syndrome) and oral stomatitis [9]. Topical uracil has been applied clinically and has prevented “Hand-Foot” syndrome from 5-FU treatment [16]. Systemic adenine has been used clinically as well in other contexts. Its unique toxicity, renal stones from the metabolite 2,8-dihydroxyadenine, is prevented by co-administration of allopurinol [17]. The dose of adenine required in the present context is much lower than that cited (100 mg/kg body weight). A clinical test of topical uracil applied to the mouth to prevent oral stomatitis and, with the same rationale, oral slow-release adenine to protect the GI mucosa from the effects of 5-FU treatment would be reasonable.

Tissue cell-type differences in pyrimidine metabolism, whether predominantly by salvage or by *de novo* synthesis, suggests a common aspect of tissue structure (see Fig. 5, bottom panels) with implications for 5-FU toxicity as well as oncogenesis. For normal squamous cells in the GI tract, replication occurs at the epithelial-mesenchymal interface and growth proceeds towards the surface layer with concomitant and progressive loss and release of cell components. For glandular cells there is a normal zone of replication at the base of the crypt that involves a Wnt (wingless and Int-1) gradient and the replicative signal originates with the intestinal subepithelial myofibroblasts near the muscularis mucosa of the mesenchyme [18]. Growing normally, epithelial glandular cells migrate away from the zone of replication towards the top of the crypt and the predominant *de novo* nutrient source in the GI lumen. At the top of the crypt the cells are shed.

As the first layer of epithelial cells for both squamous and columnar cell types derives nutrition from the mesenchymal layer below, the normal response of tissue to nutrient stress is a transient reversal nutrient source, such as likely occurs in wounding and ontogeny. For both squamous and columnar cells, persistent nutrient stress causes an activation of HIF1-alpha. This transcription factor is synthesized in response to anoxia or lack of glucose and results in a proliferation of new blood vessels in the underlying mesenchymal layer [19]. With dysplasia, the site of growth and replication persists. This results for both squamous and columnar cells in a tissue where the epithelial layer growth pattern is inverted. Of vital importance, the region of cell turnover disappears. Thus, a normal tissue, in which the regions of cell replication and cell turnover are separated, becomes a dysplastic tissue where all the epithelial cells replicate in response to the unattentuated proliferative signals originating from the mesenchymal layer. The consequence is that all the cells within the epithelial layer compete progressively for nutrients from the underlying vasculature, as they increase in number. This selection pressure, if sustained, is predicted to cause progressive phenotypic and genotypic evolution of the epithelial cells, as has been observed for GI glandular cells of the colon [20]. This oncogenic process may result in a breach by the epithelial cell of the basement membrane and EMT (epithelial mesenchymal transformation) in the competition for nutrients.

Support for this model of tissue growth, with a reversed direction of growth in oncogenesis comes from explanations it provides for several currently puzzling clinical and research observations. First, *H. Pylori* is a bacterial infection that involves the glycocalyx or superior aspect of gastric columnar cells [21]. Perhaps, surprisingly *H. Pylori* infection can lead to cancer. However, this bacterium uses glucose as a nutrient source and thus infection by *H. Pylori* may cause a local nutrient stress to the underlying glandular cells and result in a tissue with a reversed direction of growth and an increased cancer risk.

Second, the esophagus and the colon are much more likely than the small bowel to undergo epithelial cell oncogenesis. The present model would suggest that this could result from the fact that more nutrients are available and absorbed by the glandular enterocytes of the small bowel than either the squamous cells of the esophagus or the columnar colonic epithelial cells [22].

Third, although both portend increased risk of cancer, complete intestinal metaplasia (fully enabled absorptive enterocytes with villous projections) of the stomach is less likely than incomplete metaplasia of the esophagus to lead to epithelial cell cancer [23]. Incomplete metaplasia demonstrates a histologic pattern with goblet cells like that of the distal ileum not like that of the adjacent stomach. Consistent with nutrient stress as the cause of the increased cancer risk from incomplete metaplasia, normal squamous cells of the esophagus have a doubling time of 21 days, normal columnar cells of the stomach 12 days and normal columnar cells of the ileum only 4 to 5 days [24]. In addition the stomach, in contrast to the esophagus, is normally bathed in a high nutrient content mixture that would tend to mitigate nutrient stress [25].

The cellular mechanism that directs cell growth under nutrient stress within the GI tissue is not fully understood, although autophagy is believed to play an important role. Autophagy is currently the process of delivery of cellular cargo via the autophagasome for lysosomal degradation. Autophagy thus acts as a cellular survival mechanism under conditions of stress. Autophagy maintains cellular integrity by regenerating metabolic precursors and clearing subcellular debris [26, 27]. We postulate autophagy may also have an apparently paradoxic, vital role in maintaining tissue integrity by either restricting cell turnover or catalyzing cell turnover depending upon nutrient adequacy. Such a role for autophagy is consistent with the activity of coiled-coil myosin-like BCL2-interacting protein (Beclin-1). Beclin-1 (atg-6) activates autophagosome formation. When doubly deleted in mice, Beclin-1 results in an abnormal ectodermal layer and early embryonic lethality [27]. A role for autophagy in maintaining tissue structure is evident in the requirement for autophagy in order to have proper salivary gland degradation in Drosophila [28].

The control of cell degradation by autophagy in the presence of nutrient adequacy may involve regulation of Beclin-1 through abrogation of Bcl-2/Bcl-Xl inhibition by both c-jun N-terminal kinase-1 (JNK-1) [29] and death associated protein kinase (DAPK) autophagic pathways. DAPK phosphorylates Beclin-1 itself with constitutive activation of autophagy and cell death in contrast to JNK1 phosphorylation of Bcl-2/Bcl-Xl [30]. As cells of GI glandular histology approach the top of the crypts, the expression of DAPK increases [30]. Cells of both normal GI squamous and glandular histology express Beclin-1. Beclin-1 expression is increased in podocytes at crypt tops by exposure to high glucose levels [30]. Beclin-1 expression decreases with progressive dysplasia [31].

Evidence for related nutrient-responsive systems that function in the normal region of cell turnover exists. In GI glandular cells, the cell detachment process is likely mediated by Ephrin ligand that is sensitive to high glucose levels, in contrast to Eph receptor in the replication zone at the crypt base that is sensitive to low glucose states [32]. High glucose levels induce bone morphogenic protein-2 (BMP-2) [33]. BMP inhibits intestinal stem cell activation and promotes intestinal cell differentiation at the top of the colonic crypt [18].

As we did with the directed application of a tissue appropriate nucleobase to prevent 5FU toxicity, it may be reasonable to test if restoring an adequate nutrient level at the normal tissue zone of turnover would also restore normal direction of cell growth and tissue autophagic function. The intervention would consist of the directed application of nutrient mix (likely including glucose) to the tissue surface of dysplastic tissues (prior, normal region of cell turnover). Monitoring the restoration of normal, upregulated Beclin-1 expression may be a means to follow the efficacy of the intervention.
